# Molecular dynamics study of plasmon-mediated chemical transformations†

**DOI:** 10.1039/d2sc06648c

**Published:** 2023-04-08

**Authors:** Xiaoyan Wu, Tammo van der Heide, Shizheng Wen, Thomas Frauenheim, Sergei Tretiak, ChiYung Yam, Yu Zhang

**Affiliations:** a Shenzhen JL Computational Science and Applied Research Institute Longhua District Shenzhen 518110 China; b Bremen Center for Computational Materials Science, University of Bremen Bremen 28359 Germany; c Jiangsu Province Key Laboratory of Modern Measurement Technology and Intelligent Systems, School of Physics and Electronic Electrical Engineering, Huaiyin Normal University Huaian 223300 China; d Beijing Computational Science Research Center Haidian District Beijing 100193 China; e Theoretical Division, Los Alamos National Laboratory Los Alamos New Mexico 87545 USA zhy@lanl.gov; f Shenzhen Institute for Advanced Study, University of Electronic Science and Technology of China Shenzhen 518000 China yamcy@uestc.edu.cn; g Center of Integrated Nanotechnologies, Los Alamos National Laboratory Los Alamos New Mexico 87545 USA

## Abstract

Heterogeneous catalysis of adsorbates on metallic surfaces mediated by plasmons has potential high photoelectric conversion efficiency and controllable reaction selectivity. Theoretical modeling of dynamical reaction processes enables in-depth analyses complementing experimental investigations. Especially for plasmon-mediated chemical transformations, light absorption, photoelectric conversion, electron–electron scattering, and electron–phonon coupling occur simultaneously on different timescales, making it very challenging to delineate the complex interplay of different factors. In this work, a trajectory surface hopping non-adiabatic molecular dynamics method is used to investigate the dynamics of plasmon excitation in an Au_20_–CO system, including hot carrier generation, plasmon energy relaxation, and CO activation induced by electron-vibration coupling. The electronic properties indicate that when Au_20_–CO is excited, a partial charge transfer takes place from Au_20_ to CO. On the other hand, dynamical simulations show that hot carriers generated after plasmon excitation transfer back and forth between Au_20_ and CO. Meanwhile, the C–O stretching mode is activated due to non-adiabatic couplings. The efficiency of plasmon-mediated transformations (∼40%) is obtained based on the ensemble average of these quantities. Our simulations provide important dynamical and atomistic insights into plasmon-mediated chemical transformations from the perspective of non-adiabatic simulations.

## Abstract 2

Heterogeneous catalysis of adsorbates on metallic surfaces mediated by plasmons has potential high photoelectric conversion efficiency and controllable reaction selectivity.

## Graphical abstract

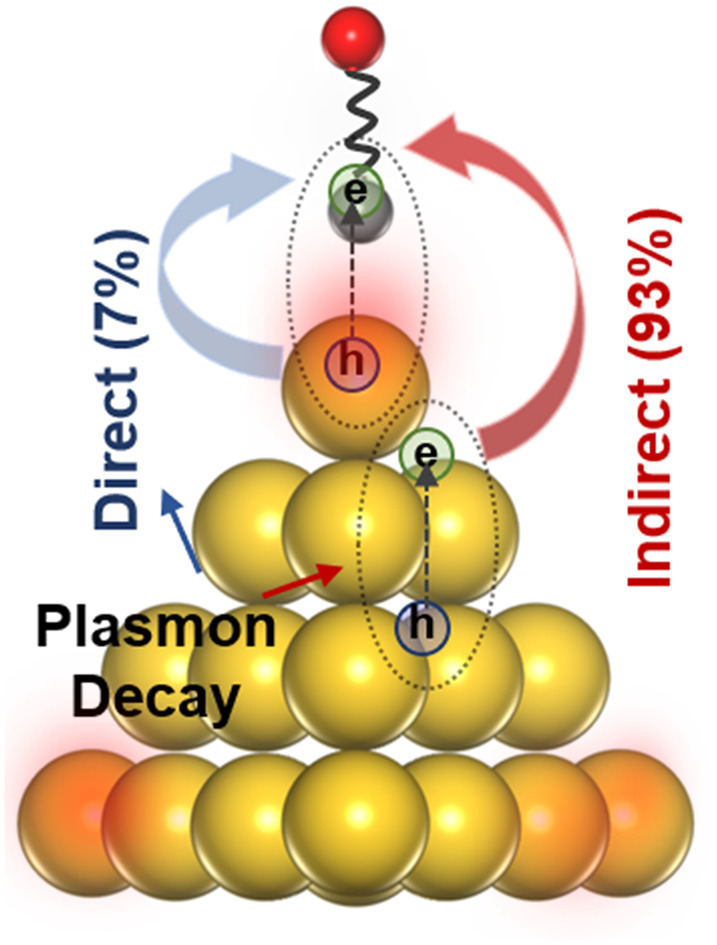

## 1 Introduction

Plasmon-mediated chemical transformations, which utilize plasmonic nanostructures as catalysts, are an emerging technology that has attracted extensive attention.^1–15^ Here, the energy of light is collected by the collective oscillations of electrons, resulting in a localized plasmon resonance (LSP), which has scattering cross-sections much larger than relevant geometric sizes.^16–18^ After being excited, LSP decays simultaneously through radiative and nonradiative pathways. Usually, the majority of the energy stored in the plasmonic field is dissipated through nonradiative decay, resulting in the formation of energetic (hot) electrons (HEs) and holes.^19^ Surface scattering, electron–phonon coupling, and electron–electron scattering are the major mechanisms that determine the distribution of these carriers.^20–23^ The energies of HEs (and their corresponding holes) are then redistributed *via* electron–electron scattering, released to phonon modes *via* electron–phonon interactions, and ultimately dissipated into the environment *via* thermal conduction.^24,25^

Thanks to their tunable optical properties, plasmons have been extensively explored in the context of driving chemical transformations *via* different mechanisms. So far, four major mechanisms have been identified in these reactions,^8^ including (1) plasmon-enhanced intramolecular excitations (or near-field effect), where the plasmon resonance overlaps with the electronic transition energies,^26–29^ (2) indirect HE transfer from metal nanoparticles (NPs) to the adsorbed molecules, where HE generation within the nanostructures is followed by a charge transfer process to the adsorbed molecules,^30–32^ (3) direct charge transfer mechanism, where the electrons are directly excited from the valence band of plasmonic materials to the virtual molecular orbital (MO) of the adsorbates,^33–36^ and (4) thermal activation (or local heating effect) due to HE relaxation.^37–39^ In particular, HE-mediated reactions have higher tunability compared to conventional temperature-driven catalysis because the energy can be selectively deposited into particular reaction coordinates in the former process.

Hence, the HE-mediated chemical transformation of adsorbates on the surface of nanoparticles induced by the unique light–matter interactions of plasmonic NPs has attracted significant attention in recent decades. For instance, the plasmonic excitations of Ag and Au NPs have been shown to activate carbonyl hydrogenation, dissociation of H_2_, reduction of nitroaromatics, *etc.*^32,40–45^ In addition, for HE-mediated processes, recent reports have suggested that interactions with semiconductors or adsorbates enhance the reaction efficiency through interfacial states.^46,47^ These interfacial states provide an additional energy dissipation pathway for the plasmons or serve as transient reservoirs of these hot carriers.^2,3,46^ Other studies have investigated the tunability of energy distribution of hot carriers to enhance quantum yield and realize the controllability of reaction pathways. For example, Manjavacas and co-workers found that small-size NPs and longer carrier lifetimes result in higher hot carrier energies but lower hot-carrier production rates, and *vice versa*.^19^ Numerous studies have reported ways of improving HE production in plasmonic nanomaterials, including the use of small NPs with large surface-to-volume ratios, designing plasmonic materials with a longer carrier mean free path, constructing hybrid nanostructures with plasmon resonances in the red and infrared regions, and designing NPs with extended plasmonic hot spots.^22–24,48^ These findings have inspired further interest in plasmonic catalysis as it offers opportunities to develop new and selective catalytic processes that were previously inaccessible.

In addition to HE-mediated reactions, the heating effect due to the HE relaxation may also activate chemical reactions. Recent debates on the thermal impact underpin the necessity of atomistic and dynamical insights *via* simulations of the HE generation, transfer, and relaxation processes on equal footing. This demands special theoretical tools such as our recent development of the NEXMD-DFTB method.^49^ Dynamic simulations of plasmon-enhanced catalysis were previously performed using different theoretical methods. For example, Meng and co-workers have investigated H_2_O splitting catalyzed by Au_20_ clusters using real-time time-dependent density functional theory (RT-TDDFT) and Ehrenfest dynamics.^50^ Their results show significant energy and spatial overlap between oscillating electron density within the Au cluster and MOs of H_2_O. Our previous work demonstrated that HEs transfer to the antibonding state of the H_2_ molecule from the metal NP upon photoexcitation. This process introduces a repulsive potential and drives the cleavage of the H–H bond.^51^ These calculations assume high-intensity photoexcitation, which depends on the strength of the external field. Recently, we also proposed a mechanism for H_2_ dissociation, which suggests that the charge transition from HE states within the metal NP to charge transfer (CT) excited states is the main channel to trigger the H_2_ reaction.^30^ Importantly, this study only involved one electron (one-hole) or mono-electronic excitation process, which facilitates understanding the basic principle of such reactions. Furthermore, this investigation explained the energy and electron transfer in terms of reaction coordinates, which elucidates the details of the reaction pathway. However, the simple model system in this study does not support plasmon excitation and therefore precludes the description of processes, including plasmon decay into hot carriers, electron transfer to adsorbed H_2_, and hot carrier decay through electron–phonon interaction.

In this work, an Au_20_ cluster is chosen, which supports plasmon-like excitation with a symmetric geometry and high stability.^52^ A CO molecule is adsorbed on Au_20_, which gives rise to CT states.^53^ This system's coupling strength between Au_20_ and CO is in the intermediate regime. So, both direct and indirect mechanisms can be studied simultaneously, together with their competition with HE relaxation. Through molecular dynamics simulations, it is found that the C–O stretching vibration is excited by HE transfers *via* CT excited states. These states are excited *via* either direct or indirect HE transfer processes. Moreover, our results reveal that both direct and indirect processes are significantly faster than the energy relaxation process in Au_20_, demonstrating that the HE transfer is the dominating process that governs the chemical transformation of the CO molecule.

## 2 Methods

### 2.1 Density functional tight-binding theory (DFTB) calculations

Geometry optimization and ground-state properties are carried out with the DFTB+ code.^54^ The auorg-1-1 parameter set, designed to describe optical excitations of organic molecules on gold nanoclusters, is employed for all computations in this work.^55^ To obtain a correct desorption reaction coordinate, the DFTB parameters associated with the repulsive potential between Au and C (O) atoms are modified (more details in the Results and discussion section). The electron occupation for each Kohn–Sham (KS) orbital is estimated by Mulliken charge analysis.^54^ Only valence electrons are considered in the DFTB method. Specifically, nine, six, and four valence electrons for Au, O, and C atoms are included. Therefore, the Au_20_–CO system has 188 KS orbitals, and 115 of them are occupied.

### 2.2 Time-dependent density functional tight-binding theory (TDDFTB) calculations

Linear response time-dependent DFTB (LR-TDDFTB) calculations within the random phase approximation (RPA) formalism are performed to obtain excitation energies, oscillator strengths, and coefficients associated with the contribution of a given KS orbital transition of each excitation.^56,57^ The optical spectra are broadened using the Gaussian function with a half-width of 0.1 eV. The adiabatic excitation energies are obtained *via* an eigenvalue equation, *ΛF*_*I*_ = *Ω*_*I*_2*F*_I_, where *Ω*_*I*_ denotes the energy of *I*^*th*^ excitation. *Λ* is the RPA matrix with its elements in MO representation given by 
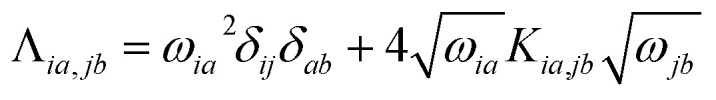
, where *ω*_*ia*_ = *ε*_*a*_ − *ε*_*i*_ and *ε*_*i*/*a*_ is the KS orbital energy. {*i*, *j*, …} and {*a*, *b*, ⋯} denote the occupied and virtual KS orbitals, respectively. *K* is the exchange-correlation kernel. Within the Mulliken approximation and LR-TDDFTB formalism, the kernel is simplified by employing *γ* approximation,^56^
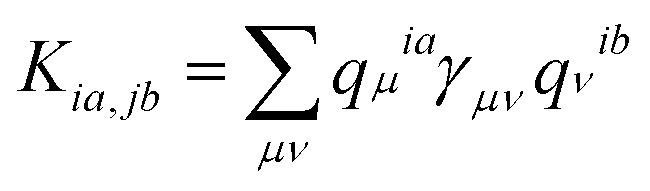
. Here *q*_*μ*_^*ia*^ denotes the Mulliken transition charges 
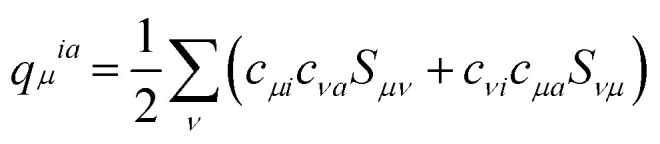
, *S* is the overlap matrix of atomic orbitals, {*μ*, *ν*, ⋯} denote atomic orbitals, and *γ* includes the Coulomb and gradient-corrected exchange-correlation kernels. The element *F*_*ia*_ denotes the contribution coefficient for KS orbital transition from an occupied orbital *i* to a virtual orbital *a*. Hence, for the Au_20_–CO system, 8395 possible transitions are included for each excited state. Because plasmon excitation usually corresponds to a higher energy excited state, hundreds of excited states are generally required. To reduce the computational cost, only occupied-virtual MO pairs with energy differences smaller than 3.85 eV are included in the excited state calculations.

### 2.3 Trajectory surface hopping (TSH) non-adiabatic molecular dynamics (NAMD) simulations

The TSH driver within the LR-TDDFTB framework is used to simulate the NAMD of Au_20_–CO initiated by plasmon excitation. Within TSH formalism, the electronic wavefunction is propagated quantum-mechanically while the nuclei are treated classically. Basically, the electronic equation of motions (EOMs) is obtained from the time-dependent Schrödinger equation1
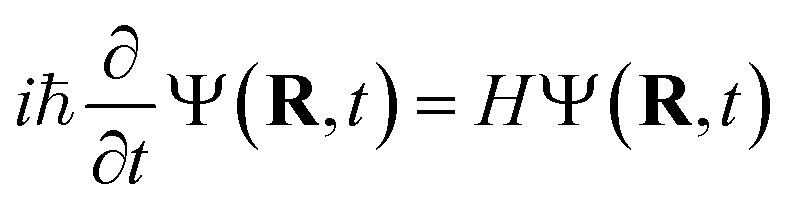
by expanding the electronic wave function *Ψ* on the basis of the adiabatic functions,2
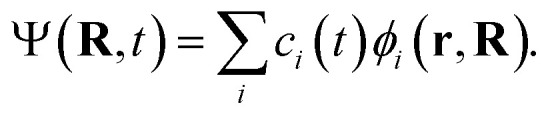


Substituting eqn (2) into eqn (1) leads to3
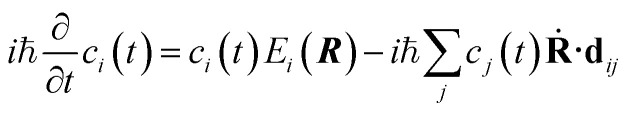
where **Ṙ**·**d**_*ij*_ is the nonadiabatic couplings. The nuclei motions are governed by Newton's equation, where the forces acting on each atom are obtained from the gradients of the adiabatic state *E*_*i*_(**R**). TSH follows the dynamics on a single trajectory but is subject to stochastic hopping between different PESs. The hopping probability *g*_*ij*_ from the electronic state *i* to some other state *j* during the nuclear time interval Δ*t* is determined jointly by the coefficient *c*_*i*_(*t*) and the time-dependent non-adiabatic coupling elements **Ṙ**·**d**_*ij*_.4
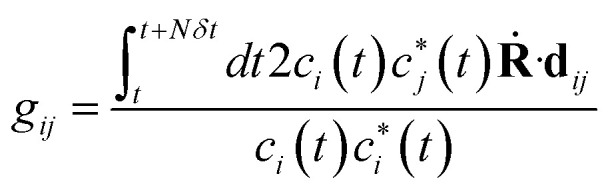
Here, 
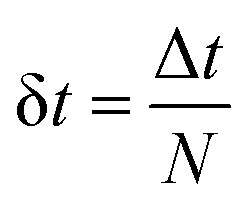
 is the time step of the electronic motion, and *N* is the number of quantum time steps (for electronic EOMs) per each classical time step for nuclei. This is recently implemented by combining the open-source software packages DFTB+ and NEXMD.^49^ The adiabatic electronic structures, including ground, excited state energies, gradients, excited state transition densities, and non-adiabatic couplings (NACs) between excited states, are described at the TDDFTB level. The non-adiabatic effects originating from coupled motions of electrons and nuclei are treated by the TSH algorithm.^49,58,59^ First, the system is optimized on its singlet ground state at the DFTB level. This is followed by a 90 ps Born-Oppenheimer ground state MD simulation with a time step Δ*t* = 0.1 fs. Here, a Langevin thermostat with a damping rate of 20 ps^−1^ is used to keep the temperature fluctuating around 300 K.^60^ After a 10 ps equilibration period, 300 snapshots of initial geometries together with nuclear velocities are sampled from the ground state trajectory with 166 fs intervals, which are used to calculate the average absorption spectrum and serve as starting points for the subsequent excited state dynamics. To obtain the average absorption spectrum, vertical excitation energies and oscillator strengths of all snapshots are computed using LR-TDDFTB.^55^ For NAMD simulations, all excited state trajectories are prepared by plasmon excitation (*i.e.*, 2.7 eV) according to the absorption spectrum and are propagated for 1 ps. The timesteps for nuclear (classical) and electronic (quantum) equations of motion are set to 0.4 fs and 0.1 fs, respectively. 160 excited states are taken into account (*N*_st_ = 160) in our simulations to ensure the inclusion of plasmon excitation and allow for possible transitions to higher excited states. As described in previous work, the instantaneous decoherence corrections (IDCs) and trivial unavoided crossings methods are adopted to improve the accuracy of calculations.^60^ The quantum timestep is further refined by a factor of 10 when potential trivial crossings are detected.

## 3 Results and Discussion

### 3.1 Geometry and electronic properties of Au_20_–CO

**Fig. 1:**
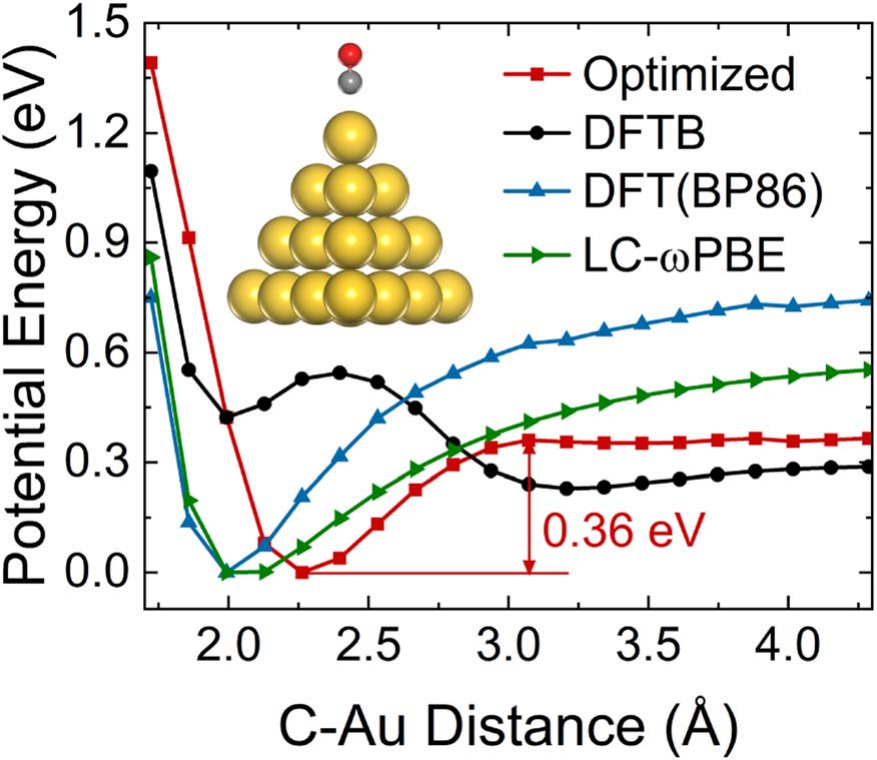
Ground state PES along the reaction coordinate of CO on Au_20_. CO molecule is placed directly above the apex site of Au_20_ and the distance with respect to Au_20_ is varied along the vertical direction. The red dotted line plots the DFTB PES from our optimized auorg-1-1 parameter set. Inset shows the ground state equilibrium structure of Au_20_–CO, where yellow, red, and gray spheres represent gold, oxygen, and carbon atoms, respectively.

Different conformations of adsorbates on the NP surface strongly influence the electronic coupling, ultimately determining the mechanism of hot carrier transfer and plasmon decay.^2,61,62^ The inset of Fig. 1 shows the ground state equilibrium geometry of Au_20_–CO optimized by the DFTB method.^55^ This structure with the CO molecule adsorbed at the apex site of the Au_20_ cluster is consistent with previous findings.^63^ To further confirm that this adsorption configuration is favorable during the dynamic process, the ground state potential energy surface (PES) along the adsorption reaction coordinate (Fig. 1) is calculated. As compared with DFT (BP86 (ref. 64)) results, it can be clearly seen that the auorg-1-1 parameter set^55^ significantly underestimates the energy barrier of CO desorption. Therefore, in order to obtain a correct topology of PES, we reoptimize the repulsive potential between atoms C (O) and Au in the auorg-1-1 parameter set by fitting the DFT ground state PES along the desorption reaction coordinate of CO on Au_20_. Specifically, the electronic part is the same as the original auorg-1-1 parameter set. The DFTB repulsive potentials, which are defined as the difference between the DFT total energy and the electronic DFTB energy, *i.e.*, (*V*_Rep_ = *E*_Total/DFT_ − *E*_Electronic/DFTB_), are represented by the following piece-wise functions,^55,65^5
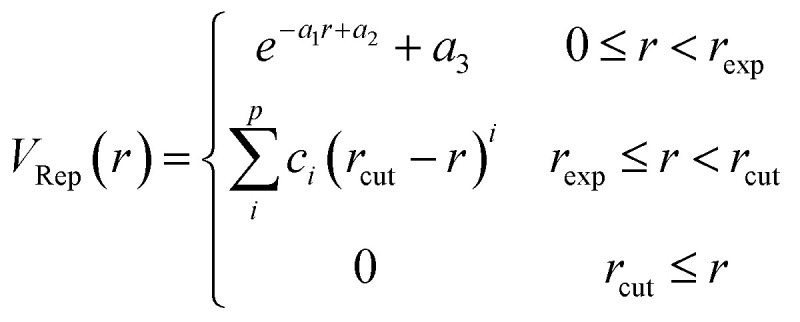
And the parameters (*r*_exp_, *a*_1_, *a*_2_, *a*_3_, *r*_cut_, *c*_*i*_) are determined from a least-squares fit into the DFT ground state PES curve along the desorption reaction coordinate of CO on Au_20_. As shown in Fig. 1, the potential profile obtained using the optimized parameter is consistent with that of DFT (BP86 (ref. 64) and LC-ωPBE^66^) results. The corresponding adsorption barrier of the CO molecule on the apex site of Au_20_ is about 0.36 eV, which is characteristic of chemisorption.

**Fig. 2:**
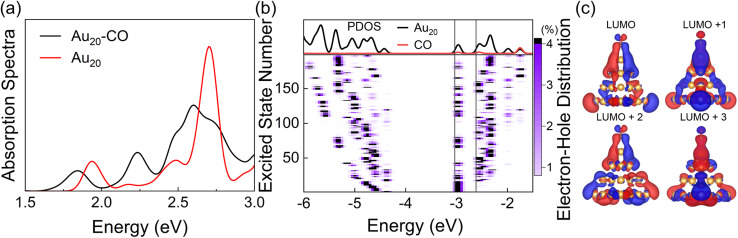
(a) Absorption spectra of Au_20_–CO and bare Au_20_ clusters obtained with LR-TDDFTB calculations. The spectra are broadened using a Gaussian function with a half-width of 0.1 eV. (b) Top panel: projected density of states (PDOSs) of Au_20_–CO. Bottom panel: electron–hole distribution of excited states. Black dashed lines indicate the position of the delocalized KS states. (c) Spatial distributions of delocalized KS MOs involved in the CT excited states. Calculations are performed based on ground-state geometry.

With the optimized DFTB parameters, we first examine the static electronic properties of plasmon excitation of Au_20_–CO. The optical absorption spectrum shows a dominant peak at 2.71 eV for a bare Au_20_ cluster (red curve in Fig. 2(a)), showing a good agreement with the value of 2.78 eV in the literature.^52^ The frequency-domain LR formalism, which describes excitations as weighted combinations of KS transitions between occupied and virtual MOs, allows an analysis of the collective character of plasmon excitation. As shown in Table S1,† the excitation energy of 2.71 eV is composed of multiple KS transitions with comparatively similar weights. The energy distributions of holes and electrons resulting from these multi-configurational transitions contain only a few peaks and have different shapes owing to the quantum confinement effect and discretized energy levels (Fig. S1†). For Au_20_–CO, the adsorption of CO induces a broadening and slightly red shift of the major absorption peak (black curve in Fig. 2(a)), which are consistent with previous studies.^61,67^ New peaks also emerge in the lower energy range of the absorption spectrum of Au_20_–CO, which are absent in the absorption spectra of bare Au_20_ and CO molecules. This signifies the presence of CT excited states following the adsorption of CO. Note that CT excited states defined here refer to excited states for which at least 0.1*e* is transferred from the Au_20_ cluster to the CO molecule upon excitation (Table S4†). In combination with the analysis of the transition component of the first excited state (S_1_, Table S3†) and the spatial distributions of MOs (Fig. 2(c)), we confirm that the KS transitions involved in the CT excited states are associated with KS MOs which are delocalized over both the Au_20_ cluster and the CO molecule. As shown in Fig. 2(c), these virtual MOs (LUMO–LUMO+3) of interest are delocalized over both CO and Au_20_ and show a significant π* character on the CO molecule. Furthermore, the electron–hole distribution of excited states (S_1_–S_200_) at the ground state geometry is depicted in Fig. 2(b). It is observed that most excited states, including the plasmon excitation state (S_116_, Fig. S1†), involve these delocalized KS MOs. (The details of calculating the electron–hole distribution are provided in the ESI.†) It is worth noting that the charge transfer from Au_20_ to CO by excitation of CT states can lead to coupling between electrons and CO vibration.^68^ Besides, we differentiate plasmon excitation from other normal excited states by examining the collectivity in excitation.^30,69,70^ Within the LR-TDDFTB or the LR-TDDFT frameworks, the collectivity of the excited state is estimated based on the eigenvector of the Casida equation. As shown in Table S2,† the S116 state, which is responsible for the absorption peak, consists of multiple single-particle excitations. Therefore, S116 is identified as the plasmon excitation.

It is well known that TDDFT calculations with local and semilocal functionals lead to spurious low-lying CT states.^71–73^ The parameterization in DFTB is however generally based on the PBE semilocal functional, which inherits this error in the description of CT states. Fig. S2† shows the absorption spectra of Au_20_ calculated with TDDFTB, TDDFT(PBE),^74^ and TDDFT(LC-ωPBE).^66^ As anticipated, both the TDDFTB and TDDFT(PBE) spectra exhibit a higher density of low-energy excited states in comparison to the long-range corrected LC-ωPBE calculation. However, the sparse set of excited states computed using the LC-ωPBE functional doesn't align with the properties of plasmon excitations. As a result, a larger cluster is required to achieve denser excited states within the LC-ωPBE framework, leading to a substantial increase in computational expenses. On the other hand, DFTB/TDDFTB with the GGA functional is more suitable for qualitatively describing the dense manifold of excited states in plasmon nanoparticles (that facilitates the HE relaxation) and the competition between HE relaxation and HE transfer-induced chemical reactions. Furthermore, the lowest excited state (S1) determined using the LC-wPBE functional also entails charge transfer from Au_20_ to CO, ensuring that the charge transfer state can be activated by plasmon excitation within the LC-wPBE framework. As a result, employing the DFTB/TDDFTB method with the GGA functional does not undermine the overall physical understanding. Therefore, in this work, we study the plasmon-like excitation dynamics of the Au_20_ systems at the level of TDDFTB. Nevertheless, for a more accurate description of excited state properties, long-range corrected DFTB can be applied, which exhibits similar accuracy to range-separated DFT methods at a significantly reduced computational cost.^75,76^

To summarize, the excited state properties of the Au_20_–CO system indicate the existence of two qualitatively different manifolds of states. One manifold of excited states is dominated by the electronic excitation confined in the Au_20_ cluster alone, which can be likened to the HE state. The other manifold involves the excitations to the delocalized hybrid Au_20_–CO MOs, which are likened to CT states.^30^ Since HE and CT states can cross, indirect HE transfer from the Au_20_ cluster to the CO molecule can occur *via* the non-adiabatic transitions between HE and CT states.^30^ Hence, direct and indirect HE transfer may contribute to the relaxation of the plasmon excitation and activation of CO vibrations. At the heart of both direct and indirect HE transfer mechanisms is the coupling between the CT states and vibrational states of the Au_20_–CO complex, which can lead HEs to dispose a portion of their energy into the vibrational motion of CO during the relaxation process. To verify this scenario and to clarify the dynamical details, including the direct/indirect HE transfer, the non-adiabatic relaxation, and the competition between the HE transfer and energy relaxation, we next perform direct NAMD simulations of plasmon excitation.

### 3.2 Real-time simulations of plasmon excitation of Au_20_–CO

**Fig. 3:**
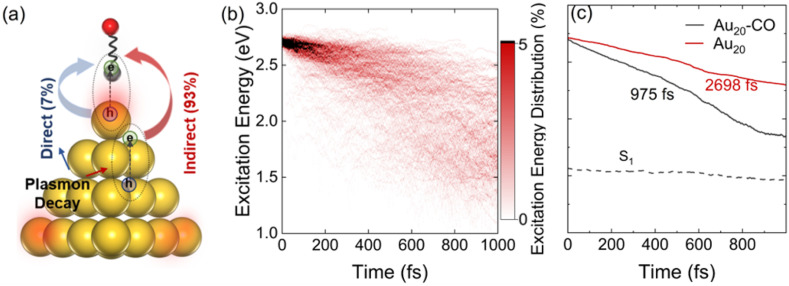
(a) Schematic of the HE transfer process in Au_20_–CO. (b) Relaxation time-energy 2D map for Au_20_–CO. (c) Evolution of the average electronic energies of Au_20_–CO and bare Au_20_. 300 trajectories have been considered for the dynamics of plasmon excitation in Au_20_–CO.

The processes of plasmon relaxation in the Au_20_–CO system are summarized in Fig. 3 where we simulated 300 trajectories in the dynamics of plasmon excitation in Au_20_–CO to obtain statistically meaningful results. The NAMD simulations allow us to delineate different processes during plasmon relaxation, as shown schematically in Fig. 3(a). To analyze the energy evolution during the relaxation process, a time-energy 2D map is constructed by calculating the probability of finding the system at a given energy level and time. Specifically, the probability is defined as6
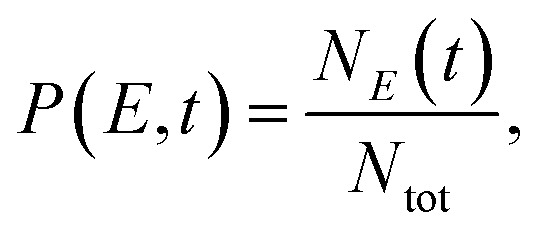
where *N*_*E*_(*t*) and *N*_tot_ stand for the number of trajectories with excitation energy *E* and the total number of trajectories, respectively. Such a representation is similar to that often used in experiments^77^ and thus provides a direct comparison between simulation and experimental data. Fig. 3(b) shows the time-energy 2D map for Au_20_–CO. The non-monoexponential decay of excitation energy and a faster relaxation compared to bare Au_20_ (Fig. S5†) indicate that multiple vibrational modes, in particular CO stretching, participate in the relaxation process of plasmon excitation. Taking the average excitation energy (*E*(*t*) = ∫*EP*(*E*, *t*)*dE*) at each time step, the lifetime of energy relaxation can be obtained by fitting the data to an exponential decay function

7 *E*(*t*) = *Ae*^−*t*/*τ*^ + *B*

where *τ* is the energy relaxation time. *A* and *B* are determined according to the conditions: *A* + *B* = *E*(0), 
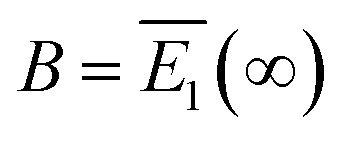
, and *E*_1_ stands for the energy of the lowest excited state (S_1_). From Fig. 3(c), the lifetime of the energy relaxation process in Au_20_–CO is estimated to be ∼1.0 ps, which is significantly faster than that of bare Au_20_ (∼2.7 ps). Here, non-adiabatic coupling between ground and excited states is not considered. Thus, the system is allowed to relax to S_1_ only. This result indicates that the hybridization of the MOs of the CO molecule and Au_20_ accelerates the energy relaxation process by offering an additional pathway for plasmon energy decay. It is postulated that the CO stretching mode participates in the relaxation process *via* the CT excited states, which accelerates the energy relaxation.

**Fig. 4:**
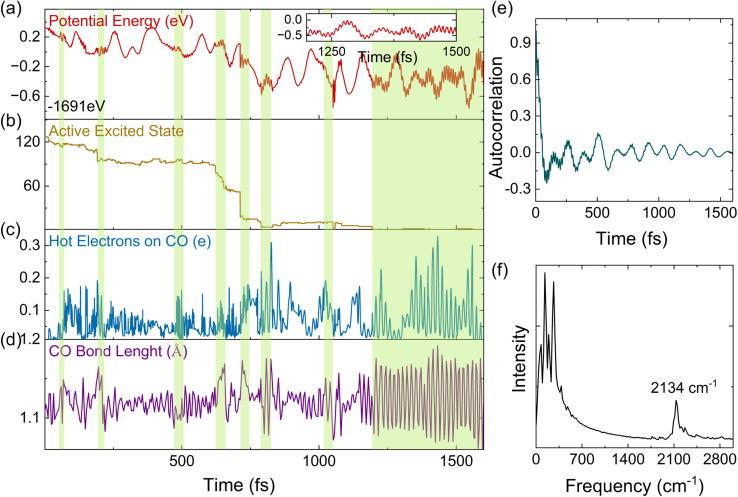
An analysis of a characteristic trajectory. From top to bottom: (a) potential energy with time, (b) active excited state, (c) transition charges on CO, and (d) variation of the bond length of CO. (e) Autocorrelation function of velocity. (f) Phonon spectra obtained by Fourier transform of (e). Inset in (a) shows an enlarged view of potential energy to highlight the high-frequency oscillations. The changes in potential energy, the activate excited state, the hot electrons on CO, and the CO bond length during the non-adiabatic hops are highlighted by the green shadows in (a–d).

Apart from the ensemble average of all the trajectories in the dynamics, each trajectory in our simulations gives insights into different possible reaction pathways. Here, we analyze a typical trajectory to shed light on the details of plasmon relaxation. Fig. 4(a–d) show the evolution of the potential energy, excited state occupations, number of HEs on the CO molecule, and the bond length of CO. The situation when the potential energy curve shows vibrations with a high frequency and small amplitude is highlighted with a green shadow. The appearance of this vibration is found to be associated with non-adiabatic hops between excited states, as shown in Fig. 4(b). The hops between excited states are accompanied by electron transfer to the CO molecule (Fig. 4(c)) and also an elongation of the C–O bond (Fig. 4(d)). Such a strong concurrent behavior across all these quantities, especially the coincidental trend between HEs on CO and the vibrational amplitude of the C–O bond length at the moment of hopping, directly reveals that the HE transfer is concomitant to the vibrational excitation of CO. From Fig. 4(c), it can be seen that HEs transfer back and forth between Au_20_ and CO repeatedly, showing energy exchange between the CO vibrational mode and the HEs. To obtain further insights into electron-vibration scattering, we identify the vibrational modes that are coupled to the electronic subsystem. For this purpose, the auto-correlation function of the velocity is calculated and plotted in Fig. 4(e). The dominant vibrational modes contributing to the electron-vibration coupling can be identified from the Fourier transform of the auto-correlation function. As shown in Fig. 4(f), it can be clearly seen that the C–O stretching mode with a frequency of 2134 cm^−1^ is excited during the relaxation process. It is noted that the chemisorption on Au_20_ leads to a red shift of stretching frequency with respect to 2395.6 cm^−1^ in the gas phase calculated by the DFTB method. On the other hand, the low-frequency vibrational modes in the range of 0–400 cm^−1^ are attributed to the Au_20_ vibrations and the vibrations induced by the Au_20_–CO coupling.

**Fig. 5:**
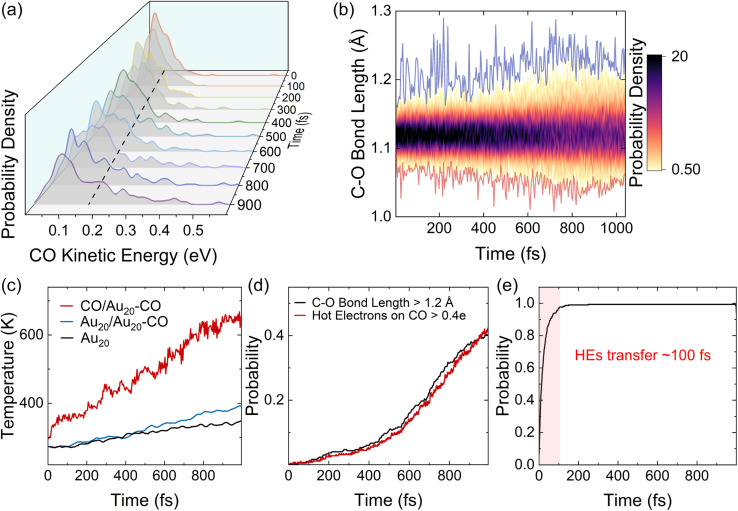
An analysis of the ensemble of trajectories. (a) Distributions of CO kinetic energy with time. (b) Distributions of the C–O bond length with time. (c) Evolution of the local temperature with time. (d) The net probability of the C–O bond length being larger than 1.2 Å and the HEs on CO being greater than 0.4*e* with time. (e) Accumulative probability of the transition charges on CO greater than 0.1*e*. All results are the statistical results averaged over 300 trajectories.

We next calculate the ensemble averages of kinetic energies, and HE distributions, to obtain experimentally relevant observables and estimate the efficiency of plasmon-induced CO activation based on these statistical results. An increase in the kinetic energy of CO is highlighted by the waterfall plots in Fig. 5(a). The average kinetic energy of CO, as marked by the dashed line, increases from 0.08 to 0.16 eV over the 1 ps relaxation timescale. Fig. 5(b) shows the evolution of the bond length of CO, where the red and blue curves represent the shortest and longest bond lengths at each time step, respectively. It can be clearly observed that the bond length of CO oscillates around 1.12 Å, and its oscillation amplitude gradually increases. We then estimate the ionic temperature of CO and Au_20_ according to the equipartition theorem (
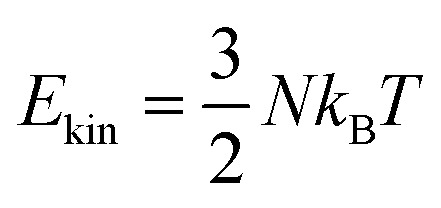
, where *k*_B_ is the Boltzmann constant and *E*_kin_ is the kinetic energy). As shown in Fig. 5(c), the temperature increase of CO is significantly faster than that of Au_20_, and the final temperature of CO is 4 times higher than that of Au_20_ after the 1 ps simulation. This steep temperature gradient strongly indicates that the activation of CO originates from HE transfer-induced vibrational excitations rather than the thermal effect. According to Fig. 5(b), we define CO to be activated if the amplitude of CO vibrational motion is larger than 1.2 Å, and the corresponding probability of CO activation is shown in Fig. 5(d). The evolution of HEs on CO shows a similar trend with the increase of the CO bond length, and the probability of activation reaches ∼40% at 1 ps, demonstrating that the HE transfer can effectively activate CO by exciting the C–O stretching mode.

Finally, we estimate the timescale of HE transfer from Au_20_ to CO by calculating the accumulated probability that HEs on CO are greater than 0.1*e* at each time step. However, the HEs on CO oscillate with time, indicating back-and-forth CT between Au_20_ and CO. In order to analyze the accumulated probability, we think that for each trajectory, once the HEs on CO are greater than 0.1*e* at a certain time, the HEs on CO at all subsequent time steps are set to 0.1*e* in the statistics. Here, the reason we choose 0.1*e* as the target value for determining whether HE transfer occurs is that the initial plasmon excitation has ∼0.1*e* charge transfer from Au_20_ to CO. As shown in Fig. 5(d) and Fig. S4,† initially, a majority of HEs are distributed on Au_20_, and the probability of direct HE transfer is only ∼7%. However, HEs rapidly transfer to CO (0.1*e*) within 100 fs. Such a fast process is also consistent with previous experimental investigations.^78^ Importantly, the energy relaxation time (∼1 ps) is significantly longer than the HE transfer timescale (∼100 fs), indicating that HE transfer occurs before electronic energy relaxation completes. Therefore, it is expected that the plasmon excitation in Au_20_ can effectively activate the CO adsorbate and mediate the subsequent chemical reactions before the nonradiative decay of HEs.

### 3.3 Conclusion

The plasmon-induced bond activation of CO adsorbed on an Au_20_ cluster triggered by HE transfer has been investigated by a non-adiabatic molecular dynamics simulation combining LR-TDDFTB theory and the TSH method. The simulations naturally treat the entire dynamical processes and multiple interactions in plasmon-mediated chemistry on an equal footing, including plasmon excitation, HE relaxation, direct/indirect HE transfer, and the activation of CO vibration mode induced by HE transfers. These dynamical processes compete with the vibration scattering process inside Au_20_ that results in local heating of the system. Our simulations provide a comprehensive picture of plasmon-induced chemistry in atomic detail.

The simulations demonstrate that CT states are crucial in plasmon-induced CO activation. The direct and indirect HE transfers are generated by the excitation of the CT states, leading to the activation of CO stretching mode, which is also an essential feature of the plasmon energy relaxation process. This activation of CO vibrational mode has been observed with high efficiency (∼40%). Importantly, the HE transfer is faster than the conventional scattering process of Au_20_. The simulations show that HE transfer from Au_20_ to CO completes within ∼100 fs, while the energy relaxation occurs on a ∼1 ps timescale.

The present simulation employs the LR-TDDFTB-based TSH method to simulate non-adiabatic molecular dynamics following the plasmon excitation directly. At each time step, 160 excited states are calculated based on the LR-TDDFTB method, pushing the limits of currently feasible theoretical methods. Nevertheless, our direct atomistic simulations outline the dynamics of the plasmon-mediated chemical transformations in terms of the evolution of the potential energy with the reaction coordinates. This enables a straightforward demonstration of the energy relaxation and HE transfers during the reaction and elucidates the details of the reaction pathways. Our numerical simulations give clear dynamical insights into CO bond activation on Au clusters. We believe that the study reported in this manuscript paves the way for simulating and understanding plasmon-mediated photochemistry.

## Data availability

The data that support the findings of this study are available from the corresponding authors upon reasonable request.

## Author contributions

The study was designed and conceptualized by YZ and CY. Simulations were performed by XW and SW. DFTB parameterization was done by XW and TH. The results were discussed and interpreted by all authors. The manuscript was written by WX and advanced by all authors.

## Conflicts of interest

There are no conflicts to declare.

## Supplementary Material

SC-014-D2SC06648C-s001
