# Supplementary material for: Molecular dynamics study of plasmon-mediated chemical transformations
Source: Chem Sci. 2023 Apr 8;14(18):4714–23. doi: 10.1039/d2sc06648c (PMC10171182; doi:10.1039/d2sc06648c)
Supplement: SC-014-D2SC06648C-s001 [file SC-014-D2SC06648C-s001.pdf]

Electronic Supplementary Material (ESI) for Chemical Science.
This journal is © The Royal Society of Chemistry 2023

Supporting Information for "Dynamical Insights into Plasmon-Mediated Chemical Transformations"

Xiaoyan Wu,[†] Tammo van der Heide,[‡] Shizheng Wen,[¶] Thomas Frauenheim,[§,‡,†] Sergei Tretiak,[∥,⊥] ChiYung Yam,[*,#] and Yu Zhang[*,∥]

†*Shenzhen JL Computational Science and Applied Research Institute, Longhua District, Shenzhen 518110, China*

‡*Bremen Center for Computational Materials Science, University of Bremen, 28359 Bremen, Germany*

¶*Jiangsu Province Key Laboratory of Modern Measurement Technology and Intelligent Systems, School of Physics and Electronic Electrical Engineering, Huaiyin Normal University, 223300, Huaian, China*

§*Beijing Computational Science Research Center, Haidian District, Beijing 100193, China*

∥*Theoretical Division, Los Alamos National Laboratory, Los Alamos, New Mexico 87545, United States*

⊥*Center of Integrated Nanotechnlogies, Los Alamos National Laboratory, Los Alamos, New Mexico, 87545, United States*

#*Shenzhen Institute for Advanced Study, University of Electronic Science and Technology of China, Shenzhen, 518000, China*

E-mail: yamcy@uestc.edu.cn; zhy@lanl.gov

Transition Mulliken Charges

The distributions of transition Mulliken charges of Ith excited state are calculated by,

$$q_A^{pq} = \frac{1}{2}\sum_{\mu\in A}\sum_{\nu} C_{\mu p}S_{\mu\nu}C_{\nu q} + C_{\mu q}S_{\mu\nu}C_{\nu p} \tag{S1}$$

$$q_A = \sum_{i\to a,b} F_{ia}^I F_{ib}^I q_A^{ab} \tag{S2}$$

where A stands for single atom in the cluster, $\{p, q \ldots\}$, $\{i, j \ldots\}$ and $\{a, b, \cdots\}$ denote the general, occupied and virtual KS orbitals, respectively. $\{\mu, \nu, \cdots\}$ denotes the atomic orbital. F^I is the eigenvector of Casida function.

Electron-Hole Distribution

Here, the distributions of holes and electrons of Ith excited state are calculated by,

$$P_{hole}(E) = \sum_{ia} w_{ia}(\Omega_I)g_i(E) \tag{S3}$$

$$P_{ele}(E) = \sum_{ia} w_{ia}(\Omega_I)g_a(E), \tag{S4}$$

where $g_i(E)$ ($g_a(E)$) is a one-dimensional (1D) broadening function for the discrete occupied i (virtual a) molecular orbitals, Ω_I is the excitation energy of Ith excited state. The Gaussian function

$$g_{i/a}(E) = \frac{1}{\sqrt{2\pi}\sigma} exp(-\frac{(E-\epsilon_{i/a})^2}{2\sigma^2}), \tag{S5}$$

where σ=0.05eV is employed. The weights $w_{ia}(\Omega_I)$ are calculated by using the eigenvector of Casida function

$$w_{ia}(\Omega_I) = (F_{ia}^I)^2, \tag{S6}$$

and satisfy the normalization condition $\sum_{ia} w_{ia} = 1$.

Table S1: Transitions for standalone Au_{20} with the highest oscillator strength for the 2.71 eV plasmon peak from the LR-TDDFTB calculation

	From	To	Weight(>0.01)
S_{128}	98	113	0.176
	102	120	0.076
	101	116	0.075
	100	119	0.070
	100	115	0.059
	81	113	0.044
	110	118	0.032
	92	113	0.022
	107	118	0.021
	95	114	0.017
	102	115	0.016
	83	113	0.016
	109	119	0.016
	106	120	0.015
	96	111	0.014
	81	112	0.013
	101	118	0.013
	92	114	0.012
	96	115	0.011
	85	111	0.011
	86	112	0.011
	108	113	0.010

Table S2: Transitions for Au_{20}-CO with the highest oscillator strength for the 2.685 eV plasmon peak from the LR-TDDFTB calculation

	From	To	Weight(>0.01)
S_{116}	104	120	0.346
	107	124	0.43
	97	117 (LUMO+1)	0.091
	96	116 (LUMO)	0.089
	115 (HOMO)	125	0.076
	105	123	0.064
	106	122	0.063
	104	121	0.009

Table S3: Transitions for Au_{20}-CO for the lowest excited state from the LR-TDDFTB calculation

	From	To	Weight(>0.01)
S_1	115 (HOMO)	116 (LUMO)	0.996
S_2	115 (HOMO)	117 (LUMO+1)	0.996

Table S4: Excitation-induced hot electrons on CO (ground state equilibrium geometry)

	CO
S_1	0.105
S_2	0.105
S_{116}	0.101

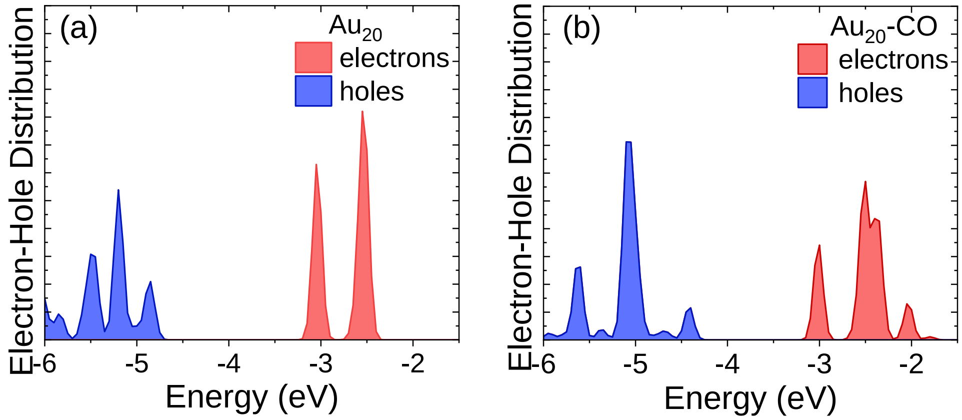

Figure S1: Electron-hole distribution of plasmon excitation state of Au_{20} and Au_{20}-CO.

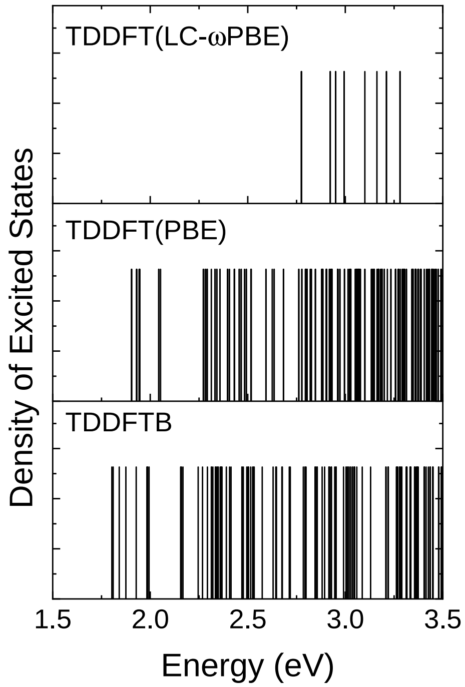

Figure S2: Density of excited states of the Au_{20} cluster calculated with TDDFTB, TDDFT with GGA functional, and TDDFT with LC functional.

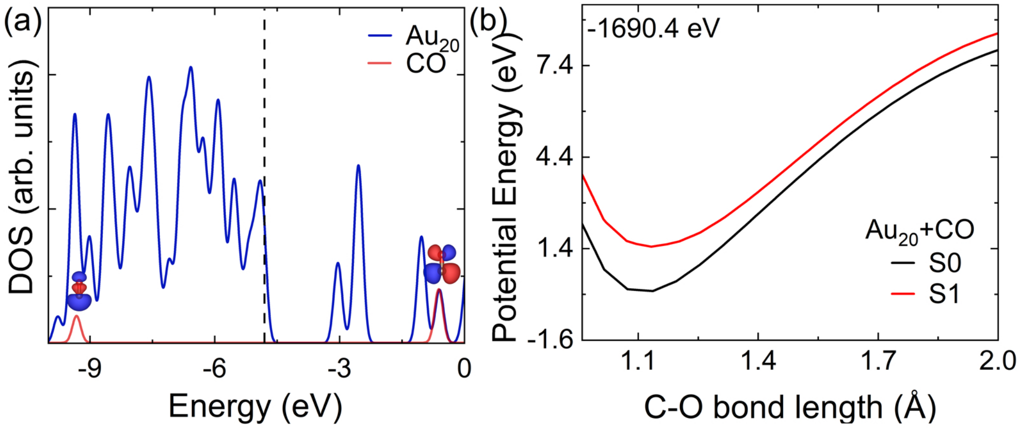

Figure S3: (a) Density of states for CO and standalone Au_{20}. (b) Potential energy for CO dissociation.

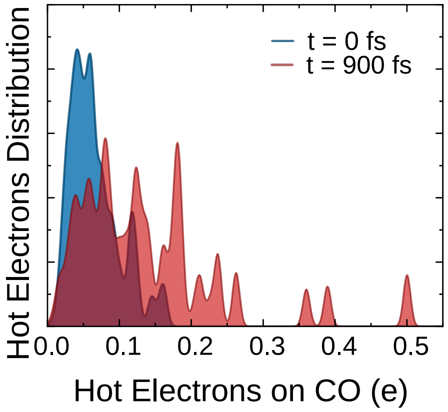

Figure S4: Distribution of the Hot Electrons on CO at 0 fs and 900 fs.

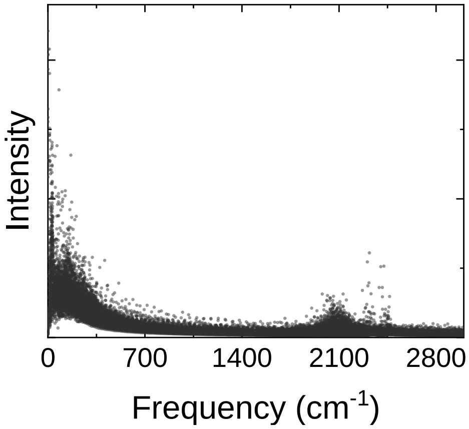

Figure S5: Spectral densities obtained by Fourier transform of the Autocorrelation functions of the velocities of Au_{20}-CO. (including all trajectories)

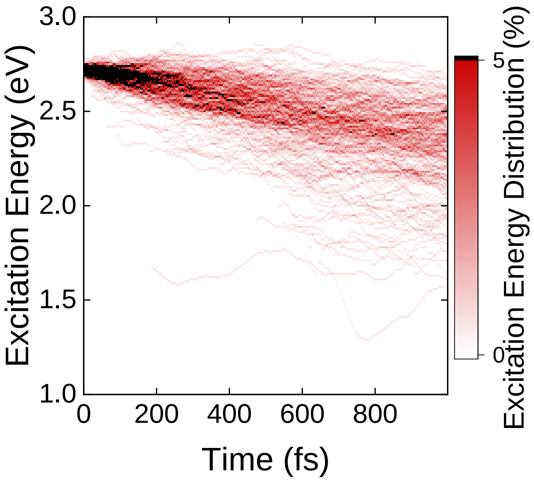

Figure S6: Relaxation time-energy 2D map for standalone Au_{20}.
